# Outcomes of Radiotherapy for Osseous Echinococcosis of Meriones meridianus

**DOI:** 10.1155/2020/6457419

**Published:** 2020-08-15

**Authors:** Chao Ma, Xuefeng Luo, Wuluhan Mahan, Yahui Tang, Yun Duan, Rui Mao, Zengru Xie

**Affiliations:** ^1^Department of Trauma Orthopedics, The First Affiliated Hospital of Xinjiang Medical University, Urumqi 830000, China; ^2^Rehabilitation Medicine Centre, The First Affiliated Hospital of Xinjiang Medical University, Urumqi 830000, China; ^3^Tumor Centre, The First Affiliated Hospital of Xinjiang Medical University, Urumqi 830000, China

## Abstract

**Background:**

Osseous cyst echinococcosis (CE) is an infectious disease that causes disability and deformity in patients, yet there is still no satisfactory treatment. Focusing on the feasibility and prognosis of radiotherapy as an adjuvant or palliative treatment for osseous CE, this study investigated the outcome of *Meriones meridianus* with osseous CE after radiotherapy.

**Methods:**

The study utilized a comparison control group design with three groups of gerbils, and 240 osseous CE gerbils were randomly divided into control, 40Gy/5times, and 50Gy/5times groups. Different doses of radiotherapy were given to the gerbils, and then, the effects of radiotherapy on gerbils and lesions were observed at 3 and 6 months after radiotherapy. Statistical analysis was done using *χ*^2^ test, unpaired *t*-test, and one-way ANOVA.

**Results:**

Significant changes (*P* < 0.05) were achieved between the three groups in terms of seven parameters at 3 and 6 months, including the number of dead gerbils and lesion sites with ulceration and infection, number of dead scolices, protein content, Ca2+ concentration, the maximum diameter of lesion site, and wet weight of cysts. Except for the number of dead gerbils and lesion sites with ulceration and infection, all other parameters were observed a big difference between 3 months and 6 months in the 50Gy/5times group.

**Conclusion:**

Radiotherapy at a dose of 50 Gy has inhibitory and therapeutic effects on osseous CE in gerbils, and radiotherapy could probably be a treatment option for persistent or recurrent osseous CE.

## 1. Introduction

As a serious zoonotic parasitosis, cyst echinococcosis has been reported worldwide. It primarily occurs in the Mediterranean Basin, the Middle East, Central Asia, Western China, the Russian Federation, Latin America, and North and East Africa [1]. Osseous CE is one of the most severe forms of the disease, and the incidence of bone involvement in CE is 0.5% to 4% of all cases of CE. The only real curative approach to osseous CE is radical surgery with complete resection of the affected bone. However, in most cases (involving the axial bone-spine, pelvic bone, and femur), bone resection is not possible due to the cyst's localization and the degree of infiltration of the cyst into the bone [2]. At present, comprehensive treatment based on surgical resection is mostly used for osseous CE treatment, and drug chemotherapy is used as adjuvant therapy. However, it is known that cure is difficult and recurrence is common. The main problem in the treatment is that osseous CE has no fibrous capsule, and then, it spreads in the bone like a “rat hole “, and there is no drug that can effectively kill it in the body. Therefore, recurrence rates are generally as high as 48% [3], and surgical treatment may also lead to disability and deformity. All the concerns above have encouraged researchers to explore more effective treatments.

Since the earliest study of CE radiation therapy published in 1927 by Dévé et al., researchers have never stopped studying radiotherapy for osseous echinococcosis. Despite the disappointing initial results, the fact that radiotherapy was parasiticidal and it was less aggressive and caused less disability and deformity than surgery encouraged researchers to conduct more studies [4]. Ionizing radiation induces DNA damage in an indirect or direct manner. The indirect effect is mediated via water radiolysis, which promotes the production of ROS (reactive oxygen species) resulting in oxidative damage, which can result in single-strand breaks. The direct effect involves direct interaction of electrons with DNA resulting in molecular distortion and double-strand breaks [5]. A study indicated that X-ray may cause an overload of intracellular calcium concentration in mitochondrial matrix by blocking calcium channels on mitochondrial and endoplasmic reticulum membranes and finally decrease the mitochondrial transmembrane potential, resulting in the pathological opening of permeability transport pores, causing abnormal endoplasmic reticulum function, mitochondrial swelling, release a certain amount of preapoptotic proteins into the cytoplasm, which can activate the caspase family, thereby inducing apoptosis in the germinal layer cells of *Echinococcus granulosus* [6]. Studies performed on rodents have shown that radiotherapy can kill or inhibit the growth of the echinococcosis and destroy the cyst [6, 7]; however, its outcome is unknown. This experiment was designed to observe the outcome of osseous CE after radiotherapy.

The successful establishment of an animal model of osseous CE is one of the bases of this experiment. Our previous experimental results showed that the subperiosteal of the hind leg of gerbils was an ideal inoculation site [8]. As one of the antigenic components in echinococcus cyst fluid, protein plays an important role in the course of the disease and is an important index to reflect the biological activity of echinococcus, and the change of its content can reflect the physiological state of echinococcus [9]. The normal echinococcosis cyst fluid contains a certain amount of Ca2+, and in echinococcosis degeneration, elevated Ca2+ content is one of the signs of improvement of CE [10]. In addition to protein content and Ca2+ concentration, changes in the wet weight and inhibition rate of cysts and some other parameters were observed in this experiment.

## 2. Materials and Methods

### 2.1. Materials

Sheep livers naturally infected with *Echinococcus granulosus* were obtained at abattoirs in and around Urumqi. The daughter cysts were aseptically dissected from the sheep liver, and the capsule skin was cut and removed. Then, they were rinsed and precipitated with 0.9% sterile saline for 3 times, and scolices were HE stained and counted, from which a 20 ml suspension containing 12 × 10^3^/L of scolices was prepared and added to 160 mg (16 × 10^4^ U) gentamicin in reserve. A total of 400 healthy female and male *Meriones meridianus* (provided by Xinjiang Institute of Endemic Disease Control, it meets the cleanliness standard of laboratory animals, and the certificate number is SCXK (Xin 2011-0003)), aged 2 to 3 months, body weight 42 ± 4 g, were involved in the study. The Coomassie protein assay kit and the calcium assay kit were purchased from Nanjing Jiancheng Bioengineering Institute; the linear accelerator was purchased from Varian, USA; and the microscope, image acquisition system, and microtome were purchased from Leica, Germany.

### 2.2. Preparation of Animal Model

200 female and 200 male gerbils were selected for the experiment, and a dose of 0.2 ml *E. granulosus* suspension obtained in 1.1 was injected under the tibia periosteum of the hind leg of each gerbil. After 12 months of inoculation, X-rays were taken to determine the bone damage at the inoculation site. Taking the definite serrated bone destruction in the tibia of gerbil as the inclusion criteria, 120 female and 120 male animal models of bone echinococcosis were selected.

### 2.3. Radiotherapy of Animal Models

According to their body weight, 240 gerbils were randomly divided into three groups: control group, 40Gy/5times group, and 50Gy/5times group, 80 in each group (each group was further divided into three-month and six-month groups, 40 gerbils per group). No treatment for the control group. Two radiation groups were treated with intermittent radiotherapy (40Gy/5times, 50Gy/5times), 5 times of consecutive radiotherapy, and another 5 times of radiotherapy after an interval of 2 days. The radiation field was designed according to the size of the lesion site by a linear accelerator, and the 6MV-X-ray source-skin radiation technique was used. The source-skin distance (SSD) of radiation was 100 cm, and the dose rate was 300 cGy/min. During radiotherapy, lead blocks were used to protect gerbils in special wooden boxes and only affected legs were irradiated.

### 2.4. General Observation

At 3 and 6 months after the radiotherapy, the number of dead gerbils and the number of lesion sites with ulceration and infection was observed.

### 2.5. Observation on the Death of the Scolex

At 3 and 6 months after radiotherapy, 15 survival gerbils were randomly selected in each group. The daughter cysts of the lesion site were aseptically took out, cut up and the cyst skin was removed, rinsed and precipitated with 0.9% sterile normal saline for 3 times, stained with eosin and observed the protoscolex under the light microscope, counting 200 scolices through the microscope, taking those who are stained or distorted and immobile as dead scolices, and calculated the number of dead scolices.

### 2.6. Detection of Protein Content and Ca2+ Concentration

According to the instructions of the Coomassie protein assay kit and the calcium assay kit, the protein content and Ca2+ concentration in the cyst fluid of each group were measured.

### 2.7. Measurement of the Lesion Site

The maximum diameter of the lesion site was measured by a compass and vernier calipers for 3 times, taking the average and recording the data.

### 2.8. Changes of Wet Weight and Inhibition Rate of Echinococcus Cysts

The echinococcus cysts were completely taken out and dried with an absorbent paper before weighed. The mean cyst wet weight between and within each group at a different time was compared, and the rate of cyst inhibition was calculated. The calculation formula for the inhibition rate between groups (%) = (*C* − *T*)/*C* × 100%, in which *C* is the mean cyst wet weight of the control group, and *T* is the mean cyst wet weight of the radiotherapy group. The inhibition rate within the group (%) = (N1 − N2)/N1 × 100%, in which N1 is the mean wet weight of cysts at 3 months and N2 is the mean wet weight of cysts at 6 months.

### 2.9. Pathological Observation

The cysts and surrounding bone tissues of each group were taken out and fixed with a formaldehyde solution, then embedded in paraffin, sectioned (slice thickness 5 *μ*m), and stained with HE, and the bone destruction and reconstruction of the lesion site were observed under the microscope.

### 2.10. Statistical Analysis

All quantitative data are presented as mean ± standard deviation (SD). For comparison between groups, one-tailed Student's unpaired *t*-test and one-way analysis of variance (ANOVA) were performed using IBM SPSS v.22.0 software and the LSD post hoc test was performed to get further results. The qualitative data were compared by the chi-squared test, and the Bonferroni correction was applied to chi-squared test for multiple comparisons. A *P* value < 0.05 was considered statistically significant.

## 3. Results

### 3.1. The Number of Dead Gerbils and the Number of Lesion Sites with Ulceration and Infection

At 3 and 6 months after the radiotherapy, the number of dead gerbils in each group gradually decreased with the increase of radiation dose. There were statistically significant differences between groups. The results of multiple comparisons showed significant differences in the comparison between the control group and 50Gy/5times group (at 3 and 6 months), as well as the comparison between 40Gy/5times group and 50Gy/5times group (at 6 months). As time goes by, the number of dead gerbils in the control group and 40Gy/5times group increased significantly. Although the number of dead gerbils in the 50Gy/5times group also increased, there was no significant difference within-group (Table 1). On the other hand, at 3 and 6 months after radiotherapy, with the increase of radiation dose, the number of lesion sites with ulceration and infection decreased gradually, and there were significant differences between groups. The results of multiple comparisons were the same as those of the number of dead gerbils. With the extension of time, the incidence of ulceration and infection of the lesion sites in the control group and 40Gy/5times group increased significantly, while that also showed an increasing trend in the 50Gy/5times group, but the within-group comparison showed no statistical significance (Table 1).

### 3.2. Death of the Scolex and Changes of Protein Content and Ca2+ Concentration in the Cyst Fluid

At 3 and 6 months after radiotherapy, with the increase of radiation dose, the number of dead scolices in each group increased significantly. The results of multiple comparisons showed significant differences in all the comparisons. However, with the extension of time, the number of dead scolices in the control group and 40Gy/5times group did not increase significantly, but that in 50Gy/5times group increased dramatically (Table 2). The protein content and Ca2+ concentration in the cyst fluid were significantly changed with the increase of radiation dose. LSD post hoc test showed significant differences in all the comparisons. The protein content and Ca2+ concentration of the control group and the 40Gy/5times group were not significantly changed over time, while obvious changes were observed in those of the 50Gy/5times group (Table 3).

### 3.3. The Changes in the Maximum Diameter of the Lesion Site and the Wet Weight of Hydatid Cysts

**Figure 1:**
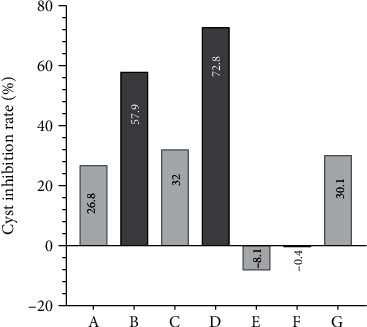
The rate of cyst inhibition. (a, b) The inhibition rate between 40Gy/5times (50Gy/5times) and control groups at 3 months. (c, d) The inhibition rate between 40Gy/5times (50Gy/5times) and control groups at 6 months. (e, f, g) Within-group inhibition rate of the control group, 40Gy/5times group, and 50Gy/5times group.

At 3 and 6 months after radiotherapy, the maximum diameter of the lesion sites of each group decreased with the increase of radiation dose, and there were significant differences between the groups. The LSD post hoc test showed significant differences in all the comparisons. With the extension of time, the maximum diameter of the lesion site increased remarkably in the control group; conversely, it decreased dramatically in the 50Gy/5times group, and no significant change was showed in the 40Gy/5times group. With the increase of radiation dose, in each group, the wet weight of the *E. granulosus* cyst decreased gradually. The LSD post hoc test showed significant differences in all the comparisons. Moreover, the wet weight of the cyst in the control group increased significantly over time; however, it decreased in the 50Gy/5times group, and no significant change was showed in the 40Gy/5times group (Table 4). These results can also be seen from the changes in the inhibition rate. As shown in Figure 1, the inhibition rate of the 50Gy/5times group was higher at 3 and 6 months; in terms of the inhibition rate within the group, the inhibition rate of the control group was -8.1%, and that of the 50Gy/5times group was the largest.

### 3.4. The Changes in the Bone Matrix of Gerbils after Radiotherapy

**Figure 2:**
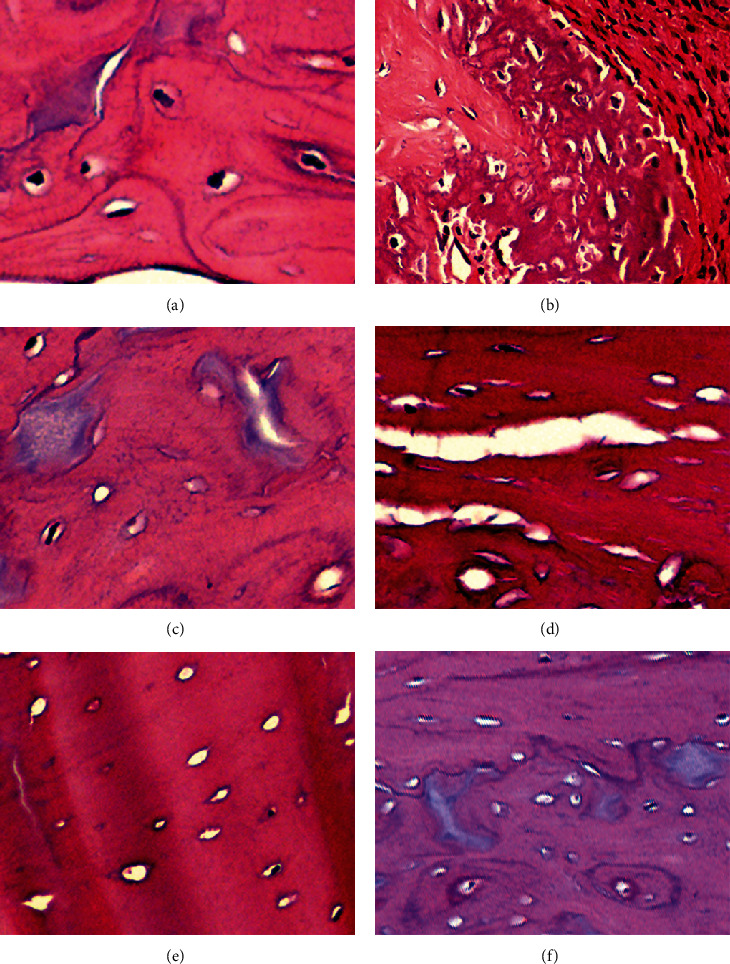
Pathological section of lesion sites. (a, b) The control group at 3 and 6 months. (c, d) The 40Gy/5times group at 3 and 6 months. (e, f) The 50Gy/5times group at 3 and 6 months.

At 3 months after radiotherapy, the bone matrix, bone lacuna, and cells in the lacuna of gerbils were normal in the control group; at 6 months, most of them were morphologically normal, and only a few cells in lacunae disappeared. In the 40Gy/5times group, at 3 months, bone matrix mucus was denatured and dissociated, and some bone cells died. At 6 months, the bone matrix was denatured and normal bone cells gradually increased. In the 50Gy/5times group, the bone matrix mucus was denatured and dissociated, and most bone cells died. At 6 months, the bone matrix was denatured and normal bone cells gradually increased (Figure 2).

## 4. Discussion

Humans are the incidental intermediate host of CE. The incidence of osseous CE is 0.5%-4%; the most common site of invasion is the spine (40%-50%), followed by large bones (25%-30%), pelvis (15%-20%) and, less commonly, the skull, sternum, scapula, and phalanges [1]. Osseous CE is one of the most severe forms of the disease. Unlike in other organs, osseous CE resembles a local malignancy; it spreads with an erosive/infiltrating pattern along the medullary and trabecular channels [11]. Although some cysts can be cured by surgery, most cases are more like an oncological disease than an infectious disease, and their diagnosis, management, and follow-up are extremely challenging. If not managed correctly, osseous CE will have a very poor prognosis in terms of long-term morbidity, comparable with that of cancer. This study was designed to further investigate the feasibility and prognosis of radiotherapy as an adjuvant or palliative therapy for osseous CE by studying the outcome of the bone echinococcosis gerbil model after radiotherapy.

As shown in Table 1, with the increase of radiation dose, the number of dead gerbils, infection, and ulceration at the lesion site of experimental gerbils in each group decreased significantly. The results of multiple comparisons showed significant differences in the comparison between the control group and 50Gy/5times group (at 3 and 6 months); however, the 40Gy/5 times group was not significantly different from the control group. This indicated that 50Gy is an effective dose. Besides, the number of deaths and infection and ulceration at the lesion site of experimental gerbils in the control group and 40Gy/5times groups were significantly increased over time. However, these increases were not obvious in the 50Gy/5times group. In the view of Xu and her colleagues, radiation-induced bone damage may occur when the bone is exposed to a dose of more than 30Gy at one time [12]. And Nadella suggests that osteoradionecrosis (ORN) affects the mandible more often than the maxilla or any other bones, it is rare after radiation of less than 60 Gy, but more common when brachytherapy is used [13]. From the above studies, it can be seen that radiotherapy inevitably causes some damage to the bone. The pathological results of this study are consistent with these conclusions; pathological characteristics of lesion sites microscopically reflect the outcome of osseous CE after radiotherapy. As shown in Figure 1, radiotherapy could cause damage to bone tissues, but part of bone tissues can repair themselves over time. This is probably related to the method and dose of radiotherapy in this study. From a radiobiological point of view, fractionated radiotherapy is significantly superior to high-dose one-time radiotherapy, so fractionated radiotherapy was used in our experiment. As no osteoradionecrosis was found, combined with the results of multiple comparisons, we speculated that 50 Gy might be a suitable radiation dose. Although radiation may have damaged bone tissues around the lesion, it still has the possibility to self-repair over time. Our results suggest that an appropriate dose of radiation can inhibit the progression of bone echinococcosis in gerbils. Therefore, it is speculated that radiation of the lesion site with an appropriate dose of radiation can effectively inhibit and even kill *E. granulosus* in it, but less damage to the surrounding tissues. Thus, as time goes on, the immune system of experimental gerbils plays a role in eliminating nonself and repairing itself, while the specific biochemical behavior needs further study.

Intracellular Ca2+ is mainly located in the endoplasmic reticulum (ER). Disruption of Ca2+ homeostasis causes dysfunction of the ER and thereby promotes apoptosis [14]. Besides, intracellular Ca2+ is an important second messenger regulating apoptosis, and disruption of Ca2+ homeostasis can induce a series of biochemical responses, which in turn lead to apoptosis [15]. It has been reported that As2O3-induced apoptosis in protoscolices could perturb intracellular Ca2+ homeostasis and activate ER stress-related apoptosis [16]. In our experiment, Ca2+ concentration in the cyst fluid and the number of dead scolices were dramatically increased with the increase of radiation dose. This is possibly because the ionization effect of radiation disrupts intracellular Ca2+ homeostasis, leading to cellular death. Ionizing radiation (IR) is an integral part of modern multimodal anticancer therapies. It has been shown IR involves the formation of reactive oxygen species (ROS) in targeted tissues, and IR further leads to an imbalance of Ca2+ by disturbing cardiocellular ROS-homeostasis [17]. Also, ionizing radiation can induce an increase in cytosolic free Ca2+ in human epithelial tumor cells. Some studies have confirmed that sustained elevations in Ca2+ can lead to chromatin degradation by Ca2+-dependent endonucleases, ultimately resulting in apoptotic cell death [18]. The mechanism by which IR disturbs Ca2+ homeostasis is complex. We believe that the increase of Ca2+ is one of the signs of improvement in echinococcosis, and a reason for such an increase is that IR can also destroy the structure of proteins and break the molecular structure of calcium-binding proteins and elevate it.

As one of the antigenic components in echinococcus cyst fluid, protein plays an important role in the course of the disease and is an important indicator of the biological activity of echinococcus [9]. Echinococcus cyst fluid consists of numerous proteinaceous and nonproteinaceous substances, which are secreted from the parasite and also absorbed from the host [19]. The functions of proteins in the cyst fluid include activating and inhibiting immune cells and inflammatory cells [20, 21], participating in the transport and assimilation of host essential lipids [22], counteracting oxidative stresses, and defending against host immune attacks [23, 24]. In *E. granulosus* cyst fluid, 153 proteins were identified, most of which were from parasitic sources. It is believed that the biological functions of the proteins in the cyst fluid are related to the unique morphology, development, and longevity of parasites in the hosts [25]. It has been shown that *E. granulosus* calmodulin is associated with exocytic activity and growth metabolism of protoscolices, indicating that calmodulin plays an indispensable role in the growth and development of *E. granulosus*, as well as in many physiological functions of *E. granulosus*. Thus, the decrease of protein content indicates that the normal physiological state of echinococcus is inhibited. In our experiment, the protein content in the cyst fluid decreased significantly with the increase of radiation dose, indicating that radiation can affect the activity of osseous CE and has a killing effect on it. As can be seen from the data in Table 2, after 50Gy/5times of radiation, the protein content, the Ca2+ concentration, and the number of dead scolices in the cyst fluid showed significant changes in experimental gerbils, suggesting that the appropriate dose of radiation tends to cure the experimental gerbils or can at least significantly inhibit the growth of osseous CE.

The changes in the wet weight of cysts and the maximum diameter of the lesion site were also observed to study the outcome of experimental gerbils under radiotherapy. As can be seen in Table 3, in the between-group comparison, with the increase of radiation dose, the maximum diameter of the lesion and the wet weight of E. granulosus cysts decreased significantly. In the within-group comparison, with the extension of time, the maximum diameter of the lesion site in the control group increased significantly, while it was dramatically decreased in the 50Gy/5times group. And no obvious pre-post differences were shown in the 40Gy/5times group. In the control group, the wet weight of E. granulosus cysts was significantly increased, and no significant change was observed in the 40Gy/5times group, while it was dramatically decreased in the 50Gy/5times group. Besides, as revealed by Figure 1, a radiation dose of 50Gy has a significant inhibition of the progression of cysts. These results showed that (a) in the control group and 40Gy/5times group, due to the absence of radiotherapy or insufficient radiation dose, the E. granulosus at the lesion site was not effectively inhibited, so that the disease continued to deteriorate, at least not effectively controlled. (b) When the radiation dose is appropriate, E. granulosus in gerbils can be effectively eliminated, and then, the immune system of gerbils can effectively eliminate the dead or dying E. granulosus.

Radiotherapy is a very important method in the treatment of malignant tumors and is also widely used in certain benign lesions. During the application and development of radiotherapy techniques, the risk of radiation-induced malignancies has been widely concerned. However, large retrospective studies have shown that no malignant transformation was observed during the use of modern radiotherapy techniques and long-term follow-up in benign bone cysts [26, 27]. This article mainly introduces the results of the application of external beam radiotherapy (EBRT) in the treatment of osseous CE in gerbils. In contrast to EBRT, brachytherapy allows the delivery of high doses of radiation by applying the radioactive source directly to the lesion [28, 29]. Currently, radioactive iodine 125, iridium 125 and 192, and phosphorus 32 are elements commonly used to treat localized bone tumors. In the musculoskeletal system, brachytherapy is mainly applied in the palliative treatment of multiple bone metastases, spinal tumors, and pelvic bone tumors, and the results are effective and promising [29–31]. At present, no studies have reported the application of brachytherapy in the treatment of osseous CE. Considering the radiosensitivity of osseous CE as well as tumor similarity, brachytherapy may be a potential palliative treatment method.

There were a few limitations to the study. Firstly, the failure rate of establishing the animal model of osseous CE is relatively high, and more effective methods need to be sought. Secondly, the specific mechanisms by which radiotherapy inhibits the progression of osseous CE remain incompletely understood.

## 5. Conclusion

In conclusion, the outcome of post radiotherapy in gerbils was evaluated by a series of parameters in our study, the results showed that an appropriate dose of radiotherapy has inhibitory and therapeutic effects on osseous CE in gerbils, which could provide theoretical support for radiotherapy as palliative therapy for osseous CE. At present, the optimal treatment for osseous CE remains surgical resection. We recommend radiotherapy as a treatment option for persistent or recurrent osseous CE. As a neglected infectious disease, osseous CE requires more effort from clinicians and researchers.

## Figures and Tables

**Table 1:** Number of dead gerbils and number of lesion sites with ulceration and infection (*n* = 40), comparison between groups (mean ± standard deviation).

Group	Number of dead gerbils				Number of lesion sites with ulceration and infection^a^			
	3 months	6 months	*χ* ^2^	*P*	3 months	6 months	*χ* ^2^	*P*
Control group	11	20	4.27	0.04	9	13	5.52	0.02
40Gy/5times group	5	13	4.59	0.03	5	10	4.30	0.04
50Gy/5times group	1^∗^	3^∗∗^^#^	1.05	0.31	2^∗^	3^∗∗^^#^	0.27	0.60
*χ* ^2^	10.4	17.4			8.57	20.35		
*P*	0.005	<0.001			0.01	<0.001		

^a^Dead gerbils were not counted. The chi-squared test and Bonferroni correction were used to detect the differences between groups. ^∗^*P* < 0.01 vs. control group; ^∗∗^*P* < 0.001 vs. control group; ^#^*P* < 0.01 vs. 40Gy/5times group.

**Table 2:** Number of dead scolices (*n* = 15), comparison between groups (mean ± standard deviation).

Group	Number of dead scolices			
	3 months	6 months	*χ* ^2^	*P*
Control group	22.40 ± 3.11	23.20 ± 2.24	0.238	0.626
40Gy/5times group	95.0 ± 5.22^∗∗^	98.20 ± 4.60^∗∗^	1.538	0.215
50Gy/5times group	135.53 ± 5.44^∗∗##^	169.33 ± 7.02^∗∗##^	236.34	<0.001
*χ* ^2^	2021.032	3207.05		
*P*	<0.001	<0.001		

The chi-squared test and Bonferroni correction were used to detect the differences between groups. ^∗∗^*P* < 0.001 vs. control group; ^##^*P* < 0.001 vs. 40Gy/5times group.

**Table 3:** Protein content andCa2+ concentration (*n* = 15), comparison between groups (mean ± standard deviation).

Group	Protein content (g/L)				Ca2+ concentration (mmol/L)			
	3 months	6 months	*t*	*P*	3 months	6 months	*t*	*P*
Control group	1.059 ± 0.055	1.088 ± 0.043	-1.58	0.12	2.802 ± 0.157	2.804 ± 0.019	-0.05	0.96
40Gy/5times group	0.733 ± 0.051^∗∗^	0.753 ± 0.034^∗∗^	-1.26	0.22	3.056 ± 0.060^∗∗^	3.068 ± 0.052^∗∗^	-0.58	0.57
50Gy/5times group	0.571 ± 0.043^∗∗##^	0.340 ± 0.032^∗∗##^	16.68	<0.001	3.546 ± 0.135^∗∗##^	3.886 ± 0.046^∗∗##^	-9.21	<0.001
*F*	372.90	1516.61			138.11	2737.03		
*P*	<0.001	<0.001			<0.001	<0.001		

Student's unpaired *t*-test and one-way analysis of variance (ANOVA) with LSD post hoc test were used to detect the differences between groups. ^∗∗^*P* < 0.001 vs. control group; ^##^*P* < 0.001 vs. 40Gy/5times group.

**Table 4:** Maximum diameter of lesion site and wet weight of cyst (*n* = 15), comparison between groups (mean ± standard deviation).

Group	Maximum diameter of lesion site (cm)				Wet weight of cyst (g)			
	3 months	6 months	*t*	*P*	3 months	6 months	*t*	*P*
Control group	2.38 ± 0.14	2.652 ± 0.05	-7.02	<0.001	3.47 ± 0.11	3.75 ± 0.31	-3.28	0.003
40Gy/5times group	1.69 ± 0.05^∗∗^	1.69 ± 0.03^∗∗^	0.09	0.93	2.54 ± 0.12^∗∗^	2.55 ± 0.08^∗∗^	-0.27	0.79
50Gy/5times group	1.40 ± 0.09^∗∗##^	1.03 ± 0.06^∗∗##^	12.90	<0.001	1.46 ± 0.07^∗∗##^	1.02 ± 0.20^∗∗##^	8.01	<0.001
*F*	372.83	4216.38			1433.46	5858.86		
*P*	<0.001	<0.001			<0.001	<0.001		

Student's unpaired *t*-test and one-way analysis of variance (ANOVA) with LSD post hoc test were used to detect the differences between groups. ^∗∗^*P* < 0.001 vs. control group; ^##^*P* < 0.001 vs. 40Gy/5times group.

## Data Availability

The data used to support the findings of this study are available from the corresponding author upon request.
